# Hospitals for the Insane

**Published:** 1846-12

**Authors:** 


ARTICLE XIII.

### HOSPITALS FOR THE INSANE.

It is gratifying to us to communicate, as it must be to every member of the profession, that rejoices in the alleviation of human suffering, to learn that Indiana has now in progress of erection a large and elegant Hospital for the Insane.

It is calculated to accommodate one hundred and forty or fifty patients, with the officers, attendants and servants necessary for its management.

The plan of its internal arrangements, made out from an examination of similar institutions, by our colleague, Dr. Evans, who is superintendent of the institution, is said to be one of the best yet devised; combining more conveniences, with less expense, than is generally to be found in such establishments. Although three hundred feet long, with contemplated additions of one hundred feet more to each end, being three and four stories high, there is scarcely a foot of room in the whole establishment that will not be usefully and appropriately occupied. The provisions for the comfort and appropriate treatment of the patients, are ample and most admirably arranged.

The system of ventillation is such, that the fire that heats, through steam pipes in a hot air chamber, all the wards in the institution, at the same time exhausts, by its draft, the rooms of their foul air and burns it.

The walls of the basement story are now put up, and in the course of the next year the building will be enclosed. So within a very short period Indiana may boast of an institution which, if well managed, will be one of the proudest monuments possible for her to erect; a monument of her benevolence; showing that her people have hearts to feel for the unfortunate, as well as heads to perceive what is her true interest.

But where stands Illinois in this matter? With a population of *two hundred and ninety-two* insane and idiotic persons in 1840, which may safely be set down at *one hundred and seventy five* insane persons at this time, what are the means provided for their care and treatment? A simple statute, by which they may if paupers enjoy the benefits of the poor laws.

However, we fain would hope that Illinois is waking up to the subject. Some time in the year 1844, Dr. Stahl, of Quincy, wrote and published a long article, calling the attention of the people to the subject. During the following winter, Dr. Mead delivered an address upon the subject to the people of Jacksonville, and we understand, will send up a petition, signed by numerous citizens of the state, praying for such an establishment, to the present session of the legislature.

And we understand that a great degree of interest in reference to the subject, has been excited in different parts of the state, during the past summer, by a visit from that celebrated philanthropist, Miss D. L. Dix, than whom, no individual has done more for the relief of the insane of America.

It was her purpose to have travelled over the whole state, visiting most of the counties, collecting facts in reference to the condition of the insane, by which course she would have been able, in a memorial to the legislature, to have set forth such an array of facts in reference to their condition, (as she has done in many of the States and in Canada,) as that to oppose provisions for the establishment of a hospital for their treatment, would have rendered a member of a legislature in a christian community, liable to the charge of gross inhumanity. But the hand of affliction arrested her labours. She has for two months been exceedingly ill, and if she is at all able to visit Springfield before the adjournment of the legislature, it will not be until toward the close of the session. We, however, confidently hope she may yet be there, as her familiarity with the subject, and her powers of persuasion, we doubt not would place the subject in such a light, before our senators and representatives, as to cause them to take some efficient action upon it.

At all events we sincerely hope they may see the matter in its true light.

A Hospital for the Insane would not only be a blessing of uncomputed magnitude to the unfortunate sufferers, who are our brethren, our sisters, and our friends, but it would be a great saving to the State, in taking better care of the insane poor, at a greatly reduced expense, who must at all events be supported by the people.

